# Early‐Season Warming Advances Autumn Senescence in Temperate Tree Saplings in Association With Early Growth Dynamics

**DOI:** 10.1111/pce.70740

**Published:** 2026-08-05

**Authors:** Yann Vitasse, Shilong Ren, Charlotte Grossiord, Matthias Saurer, Yunpeng Luo, Yan‐Li Zhang, Zhaofei Wu, Constantin Zohner

**Affiliations:** ^1^ Swiss Federal Institute for Forest, Snow and Landscape Research WSL Birmensdorf Switzerland; ^2^ Oeschger Centre for Climate Change Research University of Bern Bern Switzerland; ^3^ Big Data Research Center for Ecology and Environment, Environment Research Institute Shandong University Qingdao China; ^4^ Plant Ecology Research Laboratory PERL, School of Architecture, Civil and Environmental Engineering, EPFL Switzerland; ^5^ Forest & Soil Ecology Unit Swiss Federal Institute for Forest, Snow and Landscape Research WSL Lausanne Switzerland; ^6^ Institute for Future Initiatives University of Tokyo (UTokyo) Tokyo Japan; ^7^ BRANCH Institute Zug Switzerland

**Keywords:** growth, leaf senescence, NSC, phenology, warming experiment

## Abstract

The timing of autumn leaf senescence is driven by external cues such as temperature and photoperiod and is strongly regulated by internal developmental processes. Correlative evidence suggests that earlier budburst and warmer conditions in spring and summer could accelerate development and advance senescence, yet this hypothesis remains largely untested experimentally. We investigated how early‐season warming affects growth and senescence timing in three European tree species (*Carpinus betulus*, *Tilia cordata* and *Acer platanoides*). Saplings were grown under ambient or warmed open‐top chambers (+5°C) from January to the summer solstice, after which all saplings were maintained under identical conditions. Warming advanced leaf‐out by 14–28 days in all species. While height growth was unaffected by warming, radial growth increased significantly in *T. cordata* and *C. betulus*. Early signs of senescence (20% leaf discolouration or leaf fall) occurred significantly earlier in saplings exposed to pre‐solstice warming in *C. betulus* (−31 days) and *T. cordata* (−24 days), with effects weakening but remaining significant at later senescence stages. In contrast, *A. platanoides* showed no warming‐induced changes in growth and senescence timing. Our results suggest that early‐season warming can advance autumn senescence, and this response may be associated with species‐specific differences in early‐season growth dynamics. These findings are consistent with a potential role of growth and carbon dynamics in linking spring conditions to autumn phenology, although the underlying mechanisms remain to be directly tested. Incorporating such developmental processes into vegetation models may improve predictions of growing season changes under climate warming.

## Summary statement

Applied early‐season warming increased radial growth, changed carbon dynamics and advanced autumn senescence in *Carpinus betulus* and *Tilia cordata* saplings, but not in *Acer platanoides*. Our results suggest that faster tree development in early summer can accelerate senescence under warming.

## 1 Introduction

Plant phenological shifts in response to global warming are well documented, particularly in spring, where vegetation onset has advanced unequivocally across most extra‐tropical regions (Piao et al. 2019; Menzel et al. 2020; Büntgen et al. 2022; Vitasse et al. 2022). In contrast, autumn leaf senescence shows no consistent global trend in temperate trees (Piao et al. 2019). This inconsistency partially reflects methodological differences. Different senescence metrics capture distinct physiological and visual processes and therefore do not necessarily respond uniformly to warming. For example, the onset of leaf discolouration—rarely recorded in field observations—and the progression or end of discolouration can respond differently to warming (Zohner et al. 2023). Satellite‐based indices suggest an earlier onset of leaf discolouration across the northern hemisphere (Zohner et al. 2023). By contrast, ground observations and remote‐sensing metrics focusing on later stages (e.g., 50% or 85% greenness loss) often report no change or even delayed senescence (Gill et al. 2015; Piao et al. 2019; Menzel et al. 2020). These discrepancies are further amplified by strong interspecific variation in climate responses, even within the same stand (Panchen et al. 2015; Zuccarini et al. 2023). These disparities underscore the complexity of external and internal controls on leaf senescence and suggest that the drivers of its onset may differ from those regulating its progression, complicating attempts to identify consistent large‐scale trends under global warming.

Unlike budburst, which is generally accurately predicted by temperature and photoperiod, the onset and progression of leaf senescence are shaped by a broader set of internal and external factors, reducing the predictive power of models based solely on external cues (Vitasse et al. 2011; Garnot et al. 2025; Spafford et al. 2025). Traditionally, declining photoperiod and photosynthetically active radiation have been assumed to initiate senescence, with cooler temperatures accelerating its progression (Silvestro et al. 2025). Other external influences, such as water and nutrient availability, can also modulate its timing (Xie et al. 2018; Vitasse et al. 2021). Crucially, leaf senescence follows a sequence of developmental phases, including leaf emergence, wood growth, flowering, fruit ripening, and bud set in late summer, each altering internal states such as plant reserves and hormonal balances that can feed back on senescence dynamics. Thus, leaf senescence in temperate trees emerges from a complex interplay of endogenous developmental signals and external environmental factors (Buchanan‐Wollaston 1997; Yoshida 2003; Wingler et al. 2006; Wingler and Henriques 2022; Asad et al. 2024; Y. Luo et al. 2026).

This interplay might be orchestrated by the oxidative status of leaves and the source‐sink balance of carbohydrates (i.e., carbohydrate supply from photosynthesis versus their utilization for growth, cell maturation and development), which itself depends on the availability of key nutrients such as nitrogen and phosphorus (Wingler et al. 2006). Accordingly, non‐structural carbohydrate (NSC) concentrations in source and sink organs have been suggested as key regulators of senescence onset and progression (Wingler et al. 2006; Wang et al. 2022). Plants are thought to monitor their photosynthetic status by sensing leaf sugar concentrations and adjusting senescence accordingly (Ono et al. 2001). When carbohydrate supply exceeds sink demand, excess sugars in the source organs act as signalling molecules that can trigger the onset of senescence by modulating senescence‐associated genes (SAGs) expression and hormone signalling pathways (Wingler et al. 2006; Wingler and Roitsch 2008; Asim et al. 2022; Asad et al. 2024). Hence, sugar accumulation in mature leaves—signalling high carbon availability relative to nitrogen or other nutrients—has been proposed to advance senescence (Wingler et al. 2006). In annual plants, favourable growth conditions often increase sugar availability, stimulate growth and development and advance senescence (Wingler and Henriques 2022). In perennial plants, seasonal shifts in carbohydrate dynamics likely play an analogous role, though these mechanisms remain less well studied and characterized (Wingler and Henriques 2022; Y. Luo et al. 2026). Additionally, as leaves age and photosystems remain active, reactive oxygen species (ROS) accumulate, thereby hastening the progression of leaf senescence, especially under high solar radiation (Meier et al. 2025). Finally, any biotic or abiotic stress that alters carbohydrate supply and demand, such as frost damage, severe drought or insect outbreak, can lead to sugar accumulation or starvation; both conditions may activate chloroplast protein degradation and promote premature senescence (Bergström et al. 2026; Asad et al. 2024).

The summer solstice has been proposed as a physiological turning point marking the transition from active primary and secondary growth to tissue maturation and hardening in preparation for overwintering (T. Luo et al. 2018; Zohner et al. 2023). Around or shortly after this time—when light availability peaks—trees typically have completed most of their annual shoot elongation and a large proportion of radial growth (Etzold et al. 2022). At this point, their metabolism begins to shift towards cell‐wall strengthening and lignification, as well as the accumulation of carbohydrates and other storage compounds in reserve organs such as the stem, roots, and buds. These processes serve the development of winter cold hardiness and reserve accumulation for spring resumption, characteristics shared by all temperate deciduous trees. This post‐solstice period also coincides with reduced meristematic activity and increasing sensitivity to environmental signals such as declining day length and temperature (Maurya and Bhalerao 2017; Zohner et al. 2023). Such seasonal reprogramming suggests that processes occurring before the solstice—particularly carbon assimilation, growth, and reserve accumulation—may critically influence the timing of subsequent leaf senescence. Accelerated early‐season growth may advance senescence by shifting the sink‐source balance towards excess carbohydrates earlier in the season, whereas slower early‐season growth may delay senescence by sustaining sink demand longer (Zani et al. 2020; Zohner et al. 2023). Together, these mechanisms highlight the intricate interplay between sink‐source carbon dynamics and environmental factors in regulating the timing and progression of leaf senescence in temperate trees.

In addition, growth strategy may play a significant role in shaping how leaf senescence responds to warming. Tree species differ along a continuum from determinate to indeterminate shoot growth during the growing season, reflecting differences in development and shoot extension flexibility (Baumgarten et al. 2026; Hallé et al. 1978; Kikuzawa 1984). In species with predominantly determinate growth, shoot elongation is largely preformed and constrained early in the season, potentially limiting their capacity to exploit extended or warmer late‐summer conditions. In contrast, species with more indeterminate growth may have a greater capacity for continued growth and resource acquisition later into the growing season when conditions remain favourable, thereby delaying their leaf senescence under late warming summer (Baumgarten et al 2026). Such differences in developmental strategy could influence carbon allocation dynamics under warming and ultimately modulate the timing of leaf senescence. However, the degree of growth determinacy may vary across ontogenetic stages (e.g. between juvenile and adult trees) and environmental conditions (Kozlowski and Pallardy 1997; Körner 2003), thereby complicating broad generalizations on how climate change may impact leaf senescence depending on species growth strategies.

Here, we experimentally tested these mechanisms by growing saplings of three deciduous temperate tree species—Norway maple (*Acer platanoides* L.), small‐leaved lime (*Tilia cordata* Mill.) and hornbeam (*Carpinus betulus* L.)—in actively heated open‐top chambers during spring and early summer to enhance growth and development. We specifically asked how accelerated growth before the summer solstice affects the onset and progression of leaf senescence, hypothesizing that:

1. Saplings exposed to pre‐solstice warming will leaf out earlier and exhibit higher radial and shoot growth.
2. Pre‐solstice warmed saplings will show earlier leaf discolouration and lower late‐summer chlorophyll content, reflecting advanced development and earlier onset of senescence.

## 2 Materials and Methods

### 2.1 Study Species

We studied three temperate broad‐leaved species: *A. platanoides* L., *T. cordata* Mill. and *C. betulus* L. These species were selected because they differ in spring phenology and growth strategies. *C. betulus* commonly shows relatively early leaf flushing compared to many other broad‐leaved temperate species in this region (Vitasse et al. 2014) and exhibits a more indeterminate growth strategy, producing secondary shoots during the late season (Kozlowski 1964), whereas *T. cordata* can also produce secondary shoots occasionally. By contrast, *A. platanoides* is a relatively fast‐growing species but does not typically produce pronounced secondary shoot extensions (Hallé et al. 1978). In November 2022, we obtained 48 potted trees (2–3 years old; hereafter referred to as saplings; mean height ~57–65 cm, stem collar diameter ~6–7 mm) for each species from local nurseries (see Table S1 for the provenance and initial height) and immediately repotted them into 4 L pots using commercial peat‐based container substrate (Container‐CLASSIC one Torf; Ökohum) with moderate mineral fertilization, good drainage and aeration, high water‐holding capacity, and a pH of ~6.8.

### 2.2 Experimental Design and Temperature Manipulation

To promote early summer growth and assess its effect on subsequent autumn senescence, we exposed half of the saplings to warmer conditions from 2 January to 21 June 2023, while the other half were maintained under ambient conditions. The experiment was conducted in four hexagonal open‐top glass chambers (OTCs), 3 m high, 6 m^2^ each (Grossiord et al. 2022). For each species, saplings were randomly assigned to the four OTCs, ensuring a balanced design with 12 individuals per species and OTC. Two OTCs were maintained at 5°C above ambient air temperatures using electric heating units. Two others, also each containing 12 saplings per species, served as controls and were not actively warmed. We refer to the treatments as ‘OTC control’ and ‘OTC warming’, respectively.

A 5°C warming was chosen as it represents a realistic projection of the degree of warming expected for some winters in Switzerland by the end of the century (Fischer et al. 2022). At the study site (nearest climate station, Zurich Fluntern, 555 m a.s.l., about 8 km away), the mean annual temperature has already increased by about 2.6°C since 1960, and unusual winter warm spells exceeding past temperature norms by more than 3°C have been recorded. Temperatures in the OTCs were measured under a white radiation shield at 2 m height and recorded every 10 min (Atmos 14; Meter Group Inc., Pullman, WA, USA). During the active warming in OTCs, that is, from 2 January 2023 to 21 June 2023, the mean temperature in the two treatments was 9.6°C and 9.8°C for the two control OTCs (vs. 9.1°C outside the OTCs measured ~20 m away at 2 m height under an aluminium radiation shield) and 14.7°C and 14.9°C for the two warming OTCs (Figure 1). All pots were irrigated to full soil saturation one to three times per week, depending on conditions.

**Figure 1:**
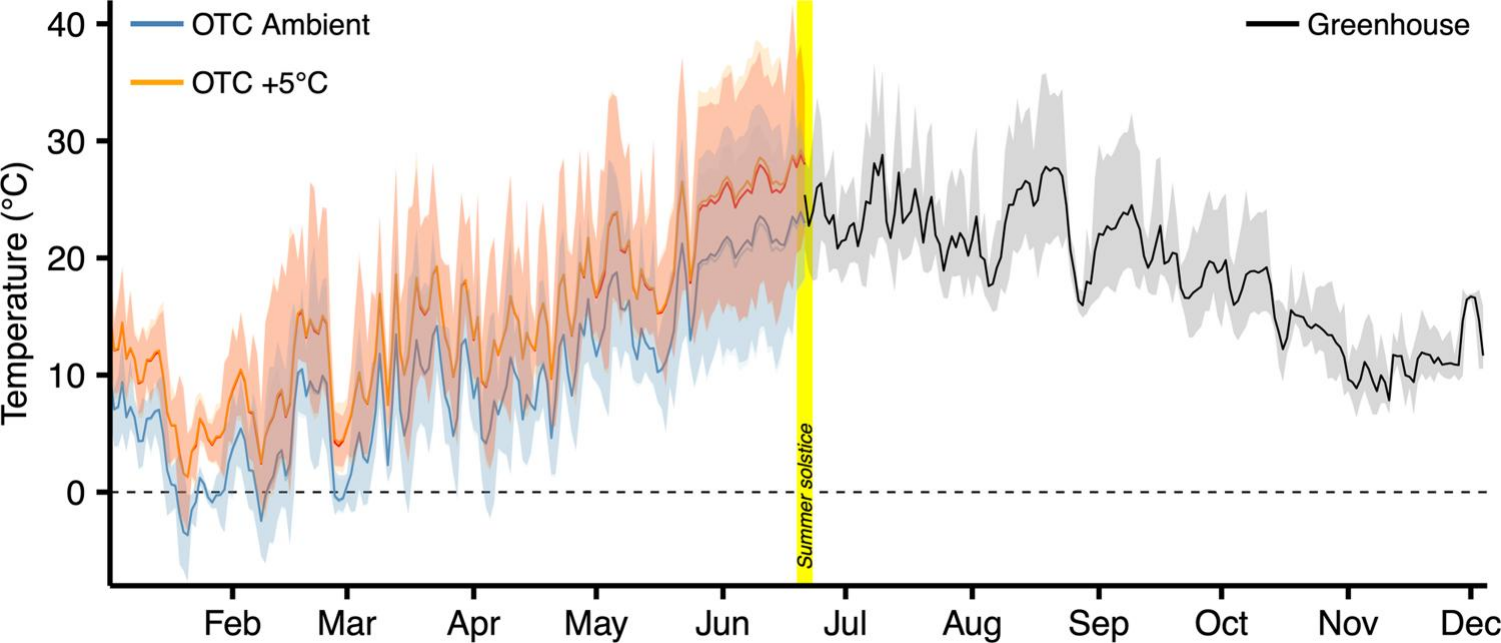
Air temperature recorded throughout the experiment. Before the summer solstice, saplings were kept in four open‐top chambers, two actively warmed (OTC + 5°C) and two at ambient temperature (OTC Ambient). After the summer solstice, all saplings were moved to a greenhouse. The lines represent daily mean air temperature, and shaded areas show daily minimum and maximum temperatures. The vertical yellow line represents the summer solstice, that is, 21st June.

Directly after the summer solstice, on 22 June 2023, we transferred all saplings from the four OTCs to a large unheated greenhouse until the end of the experiment, that is, when all leaves had either fully coloured or fallen off (beginning of December). Thus, after the summer solstice, all saplings experienced identical climatic conditions, ensuring that any difference in leaf senescence timing could be attributed to pre‐solstice conditions. In the greenhouse, saplings from each previous OTCs and species were grouped and placed in large saucers (1 m in diameter) and watered regularly. All batches of saplings were rotated randomly every 2 weeks to minimize potential microclimatic influences inside the greenhouse. The greenhouse setting also reduced potential confounding effects of direct UV exposure, strong winds, and early autumn frosts, which could otherwise induce premature leaf drop before the completion of natural senescence. Air temperature in the greenhouse was recorded at hourly intervals using a temperature logger (MX2203; Onset Computer Corp., Bourne, MA, USA) installed at plant height under an aluminium shelter.

### 2.3 Growth Measurements

Height and stem diameter (2 cm above the soil surface) of each sapling were measured three times: on 13 January 2023, that is, before leaf emergence, on 22 June 2023, that is, immediately after the transfer of all saplings to the same greenhouse, and on 7 December 2023, after all leaves had either coloured or fallen off. Height and diameter increments were calculated for the early‐summer period (13 January–22 June), during which saplings were grown under two different temperature conditions, and for the late‐summer period (22 June–7 December), when they were grown under identical conditions in the greenhouse.

### 2.4 Phenology Monitoring

Leaf emergence of every sapling was monitored twice a week from 15 February 2023 to the last stage of leaf emergence (12 May 2023). At each monitoring, the phenological stage of the most advanced bud of the sapling was recorded using a five‐stage categorical scale (Vitasse et al. 2013): Stage 0 = dormant bud (no visible development); Stage 1 = bud swelling (swollen and/or elongating buds); Stage 2 = budburst (bud scales open and leaves partially visible); Stage 3 = leaf‐out (leaves fully emerged but still folded, crinkled or pendant, depending on species); and Stage 4 = leaf unfolded (at least one leaf fully unfolded). When phenological stages were not directly observed due to rapid development between two observation dates, transition dates were linearly interpolated for each individual (Vitasse et al. 2021), resulting in an estimated temporal uncertainty lower than the observation interval (i.e., < 3–4 days).

Starting in August 2023, the same person monitored leaf senescence of every individual weekly by visually estimating the combined percentage of coloured and dropped leaves of each tree at 5% increments until 6 December 2023, when all saplings had reached 100% leaf discolouration or leaf fall. At the same time, we measured the chlorophyll content index with a chlorophyll metre (MC‐100; Apogee Instruments, Inc., USA) on three leaves per individual, focusing on the greenest leaves while avoiding shoots emerging from the roots or the youngest leaves at the top. Leaves were selected at each measurement date following this protocol and were not necessarily the same over time. This approach ensured that measurements were always conducted on fully developed, functional leaves, although it provides a conservative estimate of canopy‐level senescence, as other leaves may already have discoloured or abscised. Therefore, chlorophyll index measurements were used as a complementary metric to visual assessments of leaf discolouration and leaf fall.

### 2.5 Photosynthetic Maximum Assimilation

Using portable photosynthesis systems (LI‐6800, Li‐Cor Biosciences, Lincoln, NE, USA) equipped with a 2 cm^2^ leaf chamber, we measured leaf‐level light‐saturated photosynthesis (*A*_sat_, μmol m^−2^ s^−1^) on one mature leaf from the highest part of the crown of five randomly selected saplings per treatment (pre‐solstice warming or ambient) at four different dates: one before the summer solstice (13 June 2023), and three dates after the summer solstice, that is, on 16 August, 15 September and 9 October 2023. Individuals were selected randomly at each sampling date, so measurements, therefore, represent independent snapshots per treatment and species rather than repeated measurements of the same saplings. All measurements were conducted during sunny days. The temperature inside the cuvette was set to the corresponding ambient temperature measured inside a control OTC. Accordingly, for measurements in the heated chambers before the summer solstice, we increased the temperature in the cuvette by 5°C to match the conditions experienced by the saplings. All measurements were performed between 9:00 and 12:00, and cuvette conditions were set to constant photosynthetic photon flux density (PPFD; 1500 µmol m^−2^ s^−1^), constant CO_2_ concentration (420 ppm) and non‐limiting VPD (< 1 kPa).

### 2.6 NSC Analyses and Leaf Nitrogen and Carbon Content Around the Summer Solstice

NSCs were measured in leaves collected on 28 June 2023 and in twigs collected on 5 July 2023, that is, shortly after the summer solstice, when the saplings were moved into the greenhouse. We collected three leaves from 8 randomly selected individuals per species, and approximately 2 cm of terminal twigs (excluding buds) from 10 randomly selected individuals. NSCs were determined according to previously described protocols following Zhang et al. (2025), where NSCs are defined as low‐molecular‐weight sugars (glucose, fructose and sucrose) and starch. In detail, 10–12 mg of finely ground plant material (twig and leaves) was dissolved in 2 mL of distilled water. Afterwards, the samples were placed in a hot water bath for 30 min. Following pipetting for total NSC analysis, the extract was settled down for 20 min, after which the supernatant was collected. An aliquot of 200 μL of the transferred supernatant was added to 100 μL of Invertase from baker's yeast (Sigma‐Aldrich) to break down sucrose to fructose and glucose. Then, the samples were centrifuged (at 10 000 revolutions per minute) for 3 min to settle the solid particles at the bottom of the tube. The supernatant was enzymatically converted to gluconate‐6‐phosphate using hexokinase and phosphoglucose isomerase (from baker's yeast; Sigma Diagnostics, St. Louis, MO, USA). The total amount of glucose (free sugars) was measured photometrically at a wavelength of 340 nm using a 96‐well microplate photometer (HR 7000 Microplate Photometer; Hamilton Company, Reno, NE, USA). The total amount of NSC was measured by transferring 500 μL of the extract with solid (containing sugars and starch) at 49°C with fungal amyloglucosidase sourced from Aspergillus niger (Product Code: 10115‐5G‐F, Sigma‐Aldrich) for 15 h during the night in a water bath to digest starch into glucose. The next day, the overall NSC content was determined using the same photometric method as described above. Starch content was determined by subtracting the amount of free sugars from the total NSC. Pure solutions of starch, glucose, fructose, and sucrose were used as standard references to calibrate the NSC content of the samples. To maintain analytical consistency across runs, a standard plant powder (8.5–9.5 mg orchard leaves; Leco, St. Joseph, MI, USA) was included in each batch. NSC concentrations are reported as percentage dry matter. Samples were processed in the same laboratory using standardized protocols, and replicate analyses revealed only minor variability. NSC concentrations in leaves were determined at the individual level for all species. For *C. betulus*, twig samples for NSC analyses were pooled due to limited material availability after nutrient analyses: each replicate consisted of homogenized material from three to four individuals, resulting in three composite samples per treatment representing all sampled individuals. For *T. cordata*, most twig samples were analyzed individually; however, in five cases (3 in the control treatment and 2 in the warming treatment), material from two individuals was pooled, resulting in 7 and 8 independent replicates in the control and warming treatments, respectively (instead of 10). While pooling ensured sufficient material for analysis and captured average NSC levels at the treatment scale, it reduced the number of independent replicates. Consequently, *C. betulus* data were not analyzed statistically and are presented descriptively only. On the same leaf samples, total nitrogen and carbon concentrations were assessed. 1.0 ± 0.1 mg of dry material was weighted into tin capsules (3.3 × 5 mm, Säntis Analytical AG, Teufen, Switzerland) using a high precision balance (MT5; Mettler‐Toledo, Greifensee, Switzerland), and the samples were analyzed for leaf total C and N concentration using an elemental analyzer (iso‐Earth, Sercon, UK) coupled to an isotope‐ratio mass‐spectrometer (HS2022, Sercon, UK) with a precision of 0.1%.

### 2.7 Statistics and Analyses

No mortality was observed during the experiment, except for two *A. platanoides* individuals that were in the control OTCs. However, a few individuals were excluded in all subsequent analyses due to poor condition: two *C. betulus* (one per treatment) showing negative growth between early and late summer, and seven *A. platanoides* (five control, two warming) due to severe insect herbivory affecting leaf development and senescence assessment. We assessed the effect of pre‐solstice warming on leaf‐out date (Stage 3, commonly used as a proxy for the onset of photosynthetic activity), growth parameters (height and diameter increment), and carbon assimilation using linear mixed‐effects models fitted with the *lmer* function from the *lme4* package (v.1.1‐32). Pre‐solstice OTC was included as a random intercept to account for non‐independence among saplings within the same OTC, while species and treatments (ambient *vs*. warming) were included as fixed effects. Species‐specific treatment effects were subsequently assessed using estimated marginal means (EMMs) and pairwise contrasts from the *emmeans* package, with degrees of freedom and p‐values obtained using the Kenward–Roger approximation.

For NSC concentrations in twigs and leaves as well as leaf nitrogen and carbon content, treatment effects were evaluated using linear models including species, treatment and their interaction. A mixed‐effects model including pre‐solstice OTC as a random factor was not fitted for these variables because the number of replicates per OTC was insufficient. Consequently, individuals were pooled by treatment (warming vs. control), and the potential difference between OTCs of the same treatment could not be explicitly accounted for in the analysis. As a result, statistical analyses of NSC and leaf nitrogen and carbon content should be considered exploratory and do not provide the same level of formal treatment inference as the phenological analyses. Visual inspection of model residuals indicated that model assumptions of normality and homoscedasticity were overall satisfied.

The progress of leaf discolouration and chlorophyll index in autumn was modelled using generalized additive mixed models (GAMMs) with a Gaussian distribution using the R package *gamm4* (v.0.2‐5). For each species, the models included a smoothing spline for the DOY with four degrees of freedom, with treatment (pre‐solstice warming or ambient) as a factor modulating the spline (i.e., s(DOY, by = Treatment)). Pre‐solstice OTCs were used as a grouping variable for the random intercept to account for non‐independence among saplings within the same OTC, with individuals nested inside the OTC. The parametric treatment term in the GAMMs was used to formally test whether pre‐solstice warming significantly altered senescence dynamics, while the fitted smooths were used to derive the overall seasonal trajectory of leaf discolouration and chlorophyll loss, including estimated dates of 20%, 50% and 80% leaf colouration for each treatment. Reported uncertainty values for these thresholds correspond to 95% confidence intervals (CIs) derived from model predictions. Residual diagnostics of the GAMMS indicated some heteroscedasticity and deviations at the extremes, as expected for bounded percentage data. However, residuals were well distributed across the central range of values, within which the inferred senescence thresholds (20%, 50%, 80%) occur, supporting the adequacy of the model for describing seasonal trajectories.

## 3 Results

### 3.1 Impact of Pre‐Solstice Warming on Budburst Timing

Pre‐solstice warming resulted in significantly earlier leaf‐out in all species (Figure 2). *T. cordata* was the most affected, showing a 28.1 ± 1.8 days advance (mean difference ±SE) compared to the control, followed by *A. platanoides* (20.2 ± 2.1 days), and *C. betulus* (14.8 ± 1.8 days, Figure 2 and Table S2). Examination of OTC‐level variation revealed no systematic differences in leaf unfolding dates between the two OTCs used for the warming and ambient treatments (Figure S1 and Table S2).

**Figure 2:**
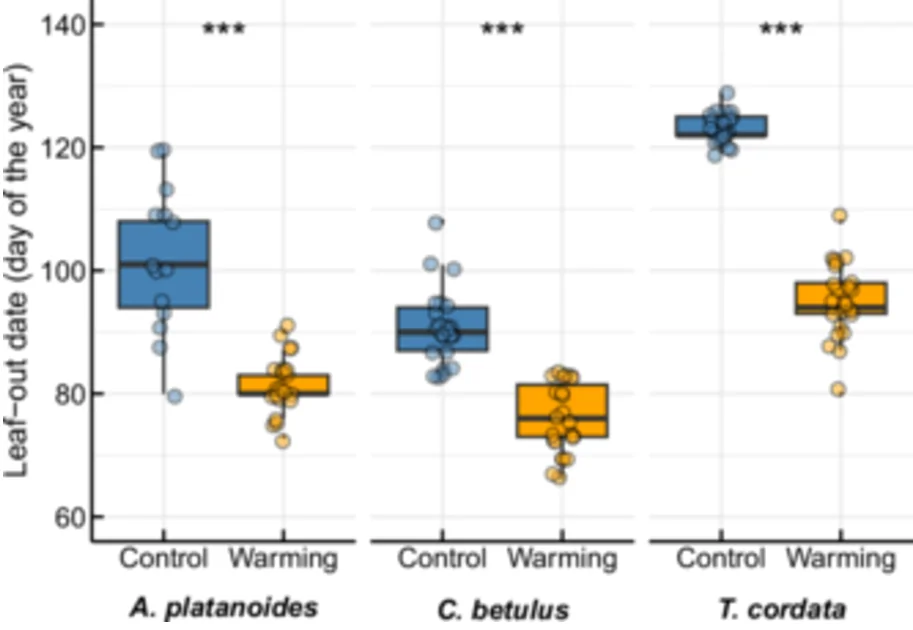
Leaf‐out timing (Stage 3) of the three species under control (blue) and warming (orange) treatments. Boxplots show the median, first and third quartiles and whiskers extending to 1.5 times the interquartile range. Statistical significance of treatment effects was assessed using linear mixed‐effects models with pre‐solstice OTCs as a random effect. ***p < 0.001. [Color figure can be viewed at wileyonlinelibrary.com]

### 3.2 Impact of Pre‐Solstice Warming on Growth Increment

Across all species, growth increment was higher before the summer solstice than after, both in height and diameter (Figure 3). Although pre‐solstice warming did not significantly affect height growth, radial growth was significantly higher under warming for *T. cordata* (+38.4%, *p* = 0.002) and for *C. betulus* (+37.8%, *p* = 0.013, Figure 3A and Table S3). After the summer solstice—when all plants were placed in a greenhouse—growth rates were low and did not differ significantly between plants previously exposed to warming or ambient conditions (Figure 3B). Remarkably, most saplings of *A. platanoides* did not show any height growth after the summer solstice, reflecting strictly shoot‐determined growth with no polycyclic shoot elongation (Figure 3B). Although only two OTCs were available per treatment, limiting the power to detect chamber‐level heterogeneity, no systematic differences in height or diameter increment were found between the two OTCs used for the warming and ambient treatments, and the variance explained by the random effect of the pre‐solstice OTCs was close to zero (Figure S2 and Table S3).

**Figure 3:**
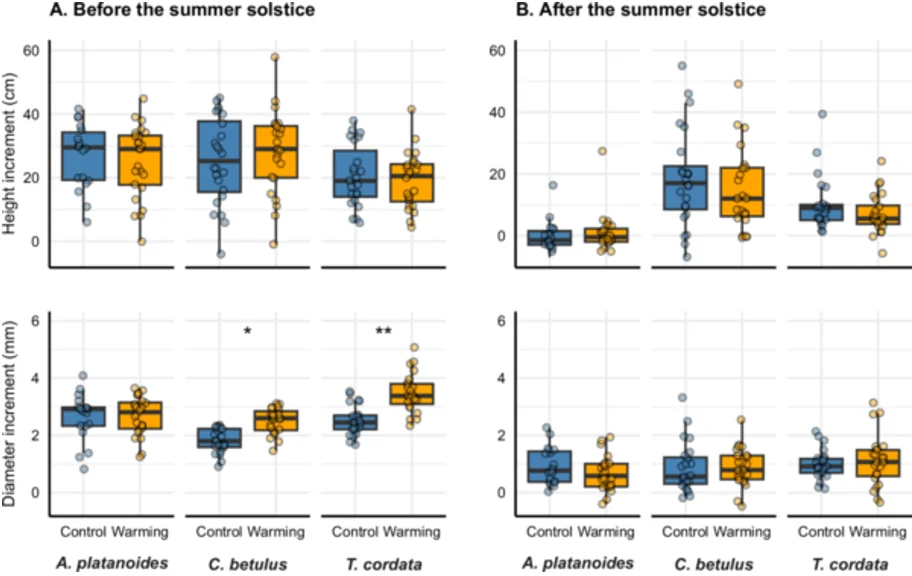
Growth increment in height and diameter before (A) and after (B) the summer solstice in control (blue) and warming (orange) treatments. Boxplots show the median, first and third quartiles and whiskers extending to 1.5 times the interquartile range. Statistical significance of treatment effects was assessed using linear mixed‐effects models with pre‐solstice OTCs as a random effect. *p < 0.05; **p < 0.01. [Color figure can be viewed at wileyonlinelibrary.com]

### 3.3 Impact of Pre‐Solstice Warming on NSCs in Twigs and Leaves

Shortly after the summer solstice, that is, after all saplings had been moved and acclimatized to the greenhouse conditions, twigs of *T. cordata* saplings exposed to pre‐solstice warming showed significantly higher starch content (+ 41.5%, *p* = 0.0013) and lower sugar content (−17.6%, *p* = 0.006) compared to those under ambient conditions (Figure 4A). For *C. betulus*, starch content in twigs appeared higher under pre‐solstice warming, but no statistical analysis was conducted on this species due to insufficient materials for NSC measurements in twigs. No significant differences were found for *A. platanoides*.

**Figure 4:**
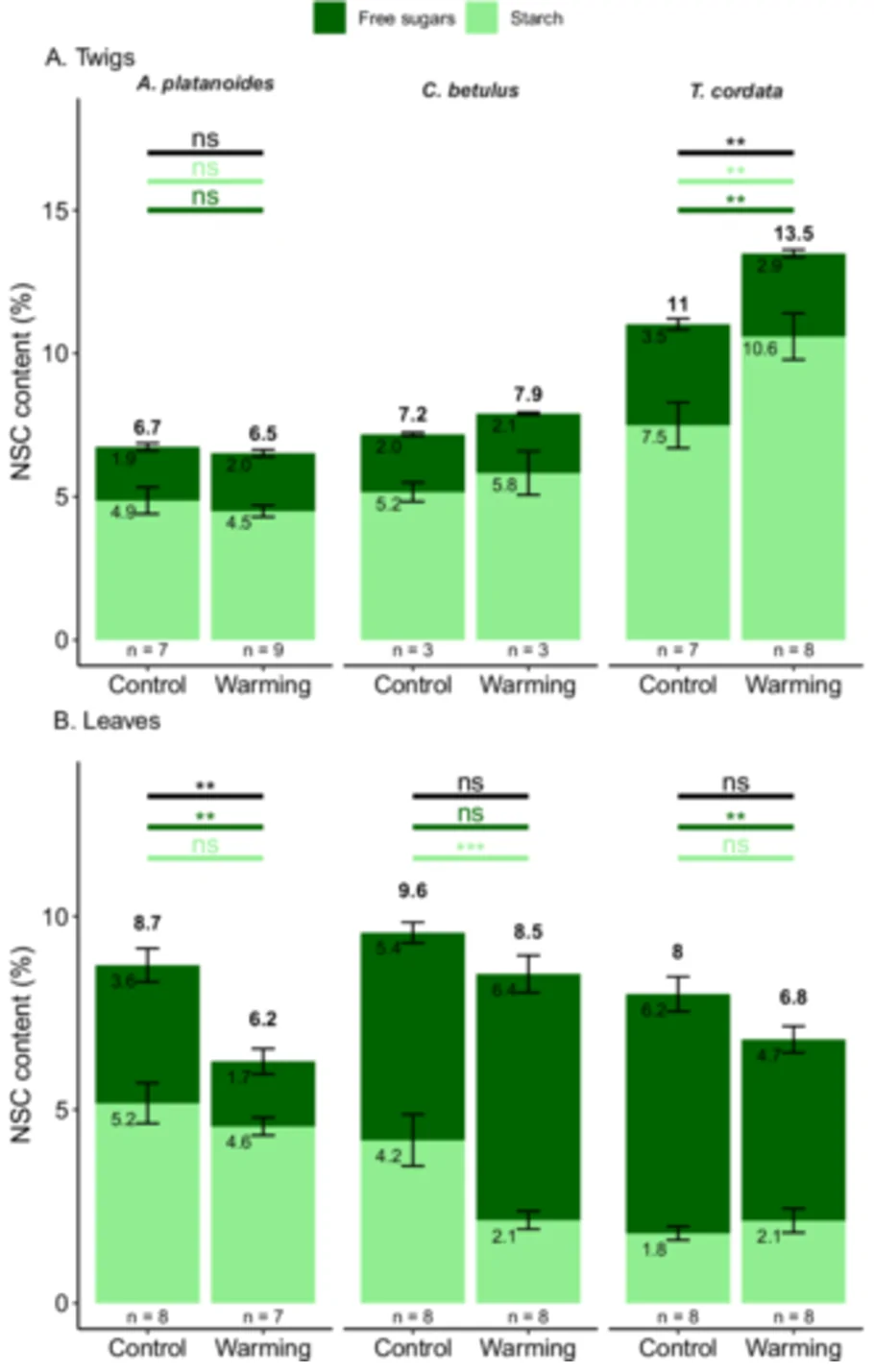
Free sugars and starch contents measured in twigs (A) and mature leaves (B) collected from the upper part of the plants shortly after the summer solstice. Values are presented as mean ± SE. Within each species and organ, dark green brackets compare free sugars between the control and warming treatments, light green brackets compare starch, and black brackets compare total NSCs. Statistical comparisons between the two treatments were performed using linear models pooling individuals within each treatment (from the two OTCs) and should therefore be interpreted as exploratory. **p < 0.01; ***p < 0.001. *n* indicates the number of individuals used per species and treatment. Note that only three measurements could be conducted on *C. betulus* for twigs due to a lack of material (pooled individuals), so this species was removed from the model for the statistical analyses. [Color figure can be viewed at wileyonlinelibrary.com]

Total leaf NSC content tended to be lower across all species in saplings exposed to pre‐solstice warming, but this trend was significant only for *A. platanoides*. This reduction in NSC content in warmed saplings was mainly due to lower sugar concentrations in *A. platanoides* (−52.9%, *p* = 0.002) and *T. cordata* (−24.3%, *p* = 0.009), whereas for *C. betulus* it was primarily associated with lower starch content (−49.2%, *p* < 0.001, Figure 4B and Table S4). Note that given the limited replication and pooling of samples, these NSC results should be interpreted as exploratory.

### 3.4 Impact of Pre‐Solstice Warming on Photosynthetic Maximum Assimilation

Maximum assimilation (*A*_max_) was similar between the two treatments for all species in mid‐June, that is, 1 week before moving the pots into the greenhouse (Figure 5). At all three measurements after the summer solstice, *A*_max_ in *C. betulus* was significantly lower in saplings previously exposed to warming (Figure 5, Table S5 and Figure S3). Specifically, *A*_max_ was reduced by 19%, 23% and 17% on 16 August, 15 September and 9 October, respectively, compared to control saplings (Figure S3). A similar, though non‐significant, pattern was observed in *T. cordata* and *A. platanoides*, where *A*_max_ was reduced by 6% and 8%, respectively, in saplings exposed to pre‐solstice warming at the last measurement in October (Figure S3).

**Figure 5:**
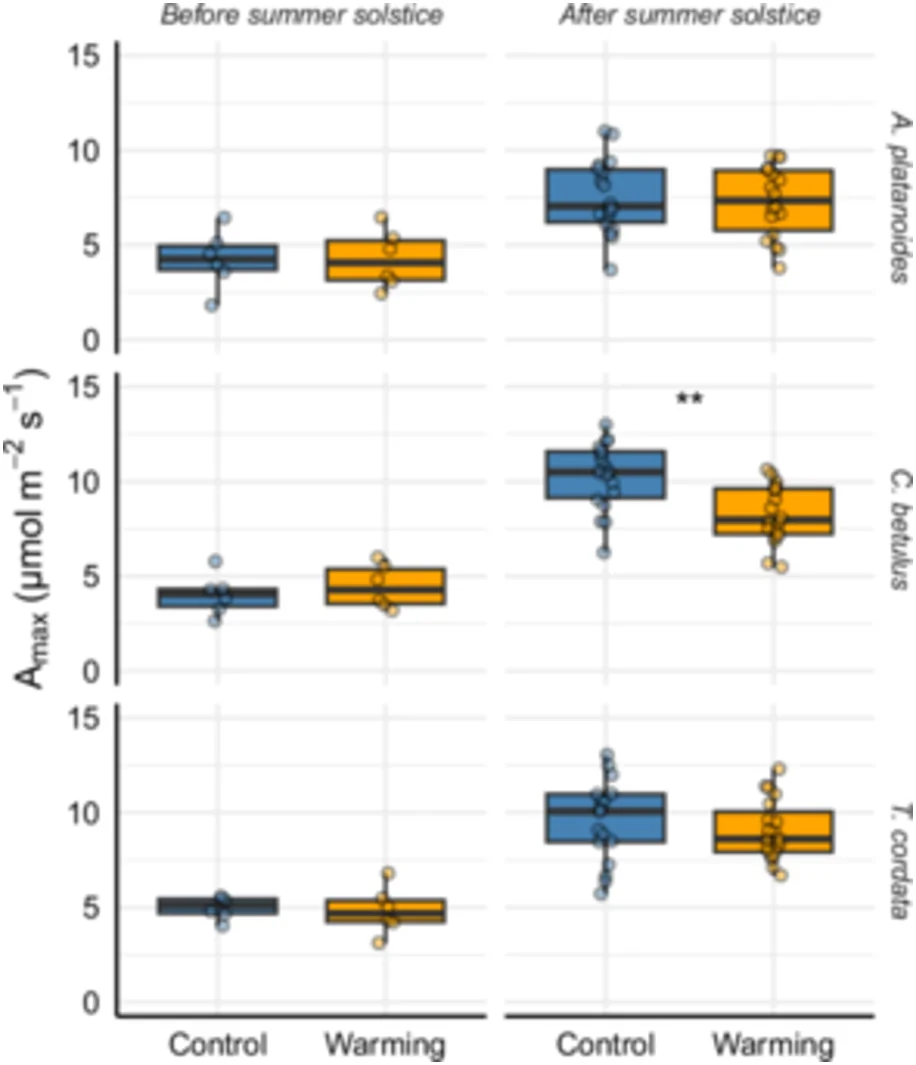
Maximum assimilation measured in the Control and Warming treatments before the summer solstice (13 June 2023) and after the solstice (three measurements in August, September, and October pooled together), when all plants were growing under identical conditions. Boxplots show the median, first and third quartiles and whiskers extending to 1.5 times the interquartile range. Note that after the summer solstice, all plants were moved into the same greenhouse; thus, any differences observed may reflect the carry‐over effects of the pre‐solstice treatment. Statistical significance of treatment effects was assessed using linear mixed‐effects models with pre‐solstice OTCs as a random effect. **p < 0.01. [Color figure can be viewed at wileyonlinelibrary.com]

### 3.5 Leaf Senescence

Pre‐solstice warming significantly accelerated leaf senescence dynamics in *C. betulus* and *T. cordata* as captured by the GAMMs models (*C. betulus*: *β* = −5.14 ± 1.18, *p* < 0.001; *T. cordata*: *β* = −4.10 ± 0.94, *p* < 0.001, Figure 6 and Table S6). These negative *β* reflect an overall shift towards lower percentages of leaf discolouration under warming across the season. For example, 20% leaf discolouration occurred on DOY 276 ± 4.2 (95% CI) in saplings exposed to warming for *C. betulus* but only on DOY 307 ± 4.4 (+31 days) in the control treatment (Figure 6). Similarly, for *T. cordata*, 20% leaf colouration was reached on DOY 280 ± 4.4 in the pre‐solstice warming treatment and on DOY 304 ± 4.2 (+ 24 days) in the control one. As leaf senescence progressed, the treatment effect declined but remained significant, with *C. betulus* reaching 50% and 80% coloration 8 and 6 days earlier under warming, respectively, while in *T. cordata* the difference was 6 and 4 days. These results suggest that the pre‐solstice warming primarily influences the onset of senescence rather than its progression. In contrast, senescence dynamics in *A. platanoides* were not significantly affected by pre‐solstice warming (Figure 6 and Table S6).

**Figure 6:**
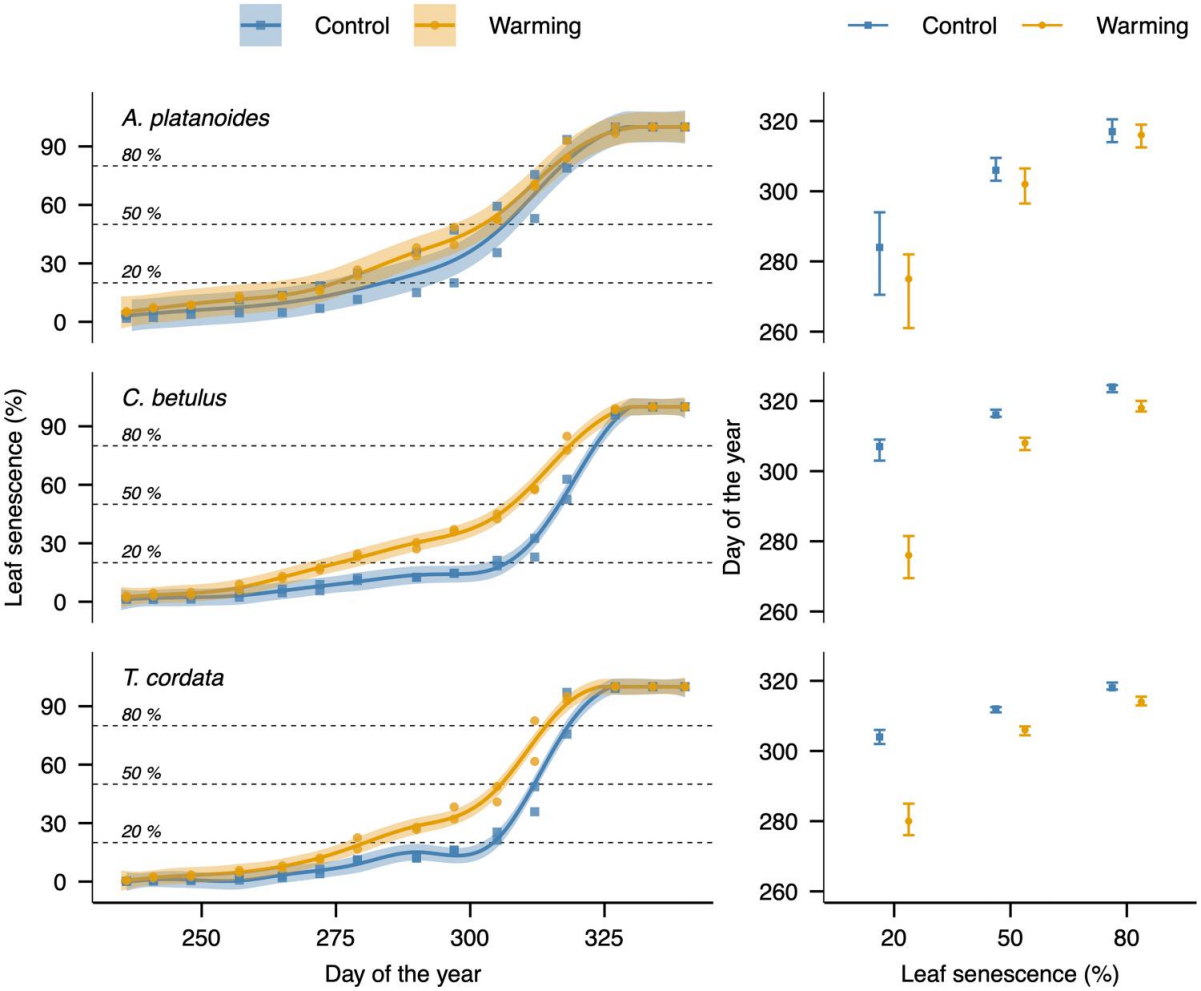
Progression of observed leaf discolouration during autumn for each species depending on the pre‐solstice treatment (control vs. warming). Left panel: dots represent mean observed values at each date, and lines represent the modelled progression of leaf discolouration using mixed‐effect generalized additive models (GAMMs), with day of year as the smoothing term and pre‐solstice OTC as a random effect. Shaded areas indicate 95% confidence intervals of the GAMM's fits. Horizontal dashed lines in the left panel represent 20%, 50% and 80% of modelled leaf discolouration. Right panel: day of year corresponding to 20%, 50% and 80% modelled leaf discolouration, with associated 95% confidence intervals derived from the GAMMs. *n* = 17–24 per species and treatment at each monitoring date. [Color figure can be viewed at wileyonlinelibrary.com]

The chlorophyll index showed similar patterns, with stronger treatment differences observed for intermediate stages of leaf discolouration in *C. betulus* and *T. cordata* (Figure S4). GAMM analysis detected a significant overall pre‐solstice warming effect for *T. cordata* (*β* = 0.97 ± 0.3, *p* < 0.001), whereas the effect for *C. betulus* was marginally significant over the full monitoring period (*β* = 0.56 ± 0.3, *p* = 0.057; Table S6). However, treatment differences on chlorophyll index were most pronounced at intermediate senescence stages, consistently with leaf discolouration observations. For example, at the date of 50% discolouration in the warming treatment, which was reached on DOY 308, and 306 for *C. betulus and T. cordata*, the chlorophyll index was 22.2% and 20.4% lower in saplings that experienced pre‐solstice warming compared to the control (Figure S4). Consistent with visual leaf discolouration patterns, the GAMM analysis showed no significant treatment effect for *A. platanoides* (*β* = 0.88 ± 0.5, *p* = 0.071; Table S6). Although chlorophyll index values tended to be lower in the pre‐solstice warming treatment at the beginning of the monitoring period (i.e., before significant changes in chlorophyll index or leaf discolouration occurred), this difference was not statistically significant, as confidence intervals overlapped (Figure S4). No significant treatment differences were detected in *A. platanoides* during the progression of leaf discolouration (Figure S4).

## 4 Discussion

Our results show that warmer temperatures from winter to early summer strongly advanced budburst timing of all three species and enhanced radial growth increment of *T. cordata* and *C. betulus*. Warmer temperatures during the pre‐solstice period also led to earlier chlorophyll degradation and leaf discolouration in autumn in these two species, but not in *A. platanoides*. This suggests that faster early‐season development—rather than the onset of photosynthesis (using bud burst as a proxy)—may promote earlier senescence, which is consistent with emerging studies highlighting a potential role of carbon source–sink balance in regulating the onset of leaf senescence timing in temperate trees (Zohner et al. 2023; Luo et al. 2025).

### 4.1 Spring Conditions Influence the Onset of Autumn Senescence

Ground‐based observations and experiments have shown that budburst timing and the duration between early phenophases often correlate with later phenophases (Fu et al. 2014; Keenan and Richardson 2015; Ettinger et al. 2018; Zani et al. 2020). Similarly, satellite‐based studies report global‐scale ‘carry‐over’ effects in temperate areas, where earlier spring growth correlates with an earlier seasonal peak and senescence onset (Keenan and Richardson 2015; Zani et al. 2020; Meng et al. 2024; Yu et al. 2025; Wu et al. 2026). However, it remains unclear whether the carry‐over effect of spring phenology on the timing of leaf senescence simply reflects a shift of the whole phenological cycle (i.e., leaves having a largely predetermined lifespan), or whether earlier spring phenology enhances development, growth, and NSC reserve accumulation, which in turn accelerates growth cessation and promotes earlier senescence via physiological mechanisms (Keenan and Richardson 2015; Zohner et al. 2023). Interestingly, in our study, *A. platanoides* showed earlier budburst under warming, similar to the other species, but did not exhibit higher height and radial growth, nor altered senescence under pre‐solstice warming. In contrast, *C. betulus* and *T. cordata* showed both increased radial growth and earlier onset of autumn leaf discolouration under pre‐solstice warming. This divergence between species suggests that advanced senescence may be more closely associated with accelerated growth and development than with budburst timing per se, potentially reflecting earlier tissue maturation and carbon reserve refilling.

### 4.2 Warmer Pre‐Solstice Conditions May Accelerate Plant Development, Reserve Accumulation and Thereby Contribute to Earlier Senescence

Under non‐limiting resources (sufficient water, light and nutrients) and no abiotic stress such as drought, warmer pre‐solstice conditions may accelerate the entire phenological cycle of temperate trees by stimulating spring growth and the replenishment of NSC reserves that are typically depleted after budburst, particularly starch stored in twigs and branches (Klein et al. 2016). Elevated starch concentration has been proposed to act as a signal of reduced sink demand and may contribute to the onset of leaf senescence (Wingler et al. 2006; Dietze et al. 2014). Starch concentrations stored in branches are particularly informative because they integrate medium‐ to long‐term carbon balance between source activity and sink demand (Hoch et al. 2003). In temperate deciduous trees, starch concentrations in branch wood typically follow a consistent seasonal pattern, with high concentrations before budbreak, a pronounced depletion around budburst, followed by refilling during the early growing season and a peak around mid‐summer—often shortly after radial growth cessation—before declining again towards autumn (Zahnd et al. 2024). In our study, we found that pre‐solstice warming accelerated the post‐budburst recovery, in twigs of *T. cordata* and possibly *C. betulus* (not tested statistically due to the low number of replicates), as reflected by higher starch content around the summer solstice, whereas no difference was found in *A. platanoides*. Thus, starch concentrations in storage organs may have increased earlier in *T. cordata* and *C. betulus*, consistent with the idea that altered NSC dynamics under early‐season warming may contribute to earlier leaf senescence. However, direct evidence for the role of sugar‐sensing pathways in advancing leaf senescence remains limited, especially at the individual level, and requires further experimental investigation, including potential links to feedback downregulation of photosynthetic capacity, SAGs induction, or hormone‐mediated signalling (Rolland et al. 2006; Lim et al. 2007; Thomas 2013).

The interpretation of NSC dynamics in leaves is more complex, as leaf NSC pools fluctuate strongly on diurnal and seasonal timescales and primarily reflect short‐term source–sink dynamics rather than longer‐term carbon status (Martínez‐Vilalta et al. 2016). Nevertheless, shortly after the summer solstice, we observed overall lower leaf NSC concentrations under pre‐solstice warming across species (significant only in *A. platanoides*), with reduced soluble sugar concentrations in *A. platanoides* and *T. cordata* and lower starch concentrations in *T. cordata*. These patterns may reflect enhanced carbon export, increased respiratory costs, or higher growth demand under warming conditions, but should be interpreted cautiously given that NSC was measured only once in few replicates and may vary considerably over short‐term scales.

Lihavainen et al. (2021) girdled adult poplar clones to block phloem transport, which caused photosynthate accumulation in the crown and an earlier start of senescence, attributed to altered sink‐source balance but also C/N status. In our study, we found that pre‐solstice warmed saplings tend to have lower leaf C/N ratios (significant for *A. platanoides* and *C. betula*, Figure S5) due to higher foliar nitrogen (Figure S5) around the summer solstice compared with non‐warmed saplings. Higher foliar nitrogen under warming conditions may reflect increased nitrogen remobilization from storage to meet higher growth demand (Millard and Grelet 2010).

All these changes in resource allocation, together with faster pre‐solstice growth and reserve accumulation, may explain why pre‐solstice warmed saplings initiated senescence earlier even under identical post‐solstice conditions. However, further experiments across a broader range of species, along with more detailed quantification of carbohydrate reserves at regular intervals and in different plant organs, including roots, are needed to confirm and refine these results.

In addition, because our experimental design primarily supports inference at the treatment level, and senescence timing was derived from GAMM models fitted at that level, we could not formally test relationships between individual growth and senescence timing. Nevertheless, to further explore potential links at the individual scale, we analyzed relationships between early‐season growth (diameter increment) and phenological status at DOY 290, corresponding approximately to the onset of senescence (~20% coloration across species). In *C. betulus* and *T. cordata*, we found a significant negative correlation between diameter increment and chlorophyll index, indicating that faster‐growing individuals exhibited lower chlorophyll content at the onset of senescence (Figure S6). Consistently, for leaf colouration, the relationship with diameter increment was positive (faster‐growing individual showed higher percentage of coloured or fallen leaves) for these two species but not statistically significant (Figure S6), whereas no significant correlations were found at later DOY, both with leaf senescence or chlorophyll index. These exploratory results are consistent with the hypothesis that faster early‐season growth may contribute to earlier senescence, although they should be interpreted cautiously given the limitations of the dataset.

### 4.3 Determinate vs. Indeterminate Growth May Shape Autumn Phenological Responses to Warming

The differences in chlorophyll content between treatments already observed in late summer, before the first visible sign of discolouration (Figure S4), may be related to species‐specific growth patterns. In *C. betulus*, which exhibits a largely indeterminate shoot growth strategy, many leaves formed during pre‐solstice warming may have already begun senescing by late summer. Consequently, the chlorophyll measurements targeting the greenest leaves were likely biased towards younger, post‐solstice leaves, resulting in no significant effect between the two treatments. In contrast, *T. cordata* and especially *A. platanoides* exhibit a more strictly determinate shoot growth strategy, producing few (*T. cordata*) or no new leaves (*A. platanoides*) after the summer solstice, as evidenced by the absence or very limited height increment during this period. For these species, treatment effects were already evident in late summer, with lower chlorophyll content under the warming treatment (Figure S4), possibly reflecting earlier leaf ageing and accumulating oxidative stress associated with earlier budburst.

The degree of growth (in)determinacy may also explain why we did not observe any significant changes in either height or stem increment in *A. platanoides*, despite earlier budburst (species with a strong determinate growth strategy), whereas radial growth was enhanced in *T. cordata* (less deterministic) and in *C. betulus*, having intermediate and highly indeterminate growth strategies, respectively. These differences in growth strategies and resource allocation suggest contrasting responses between species under future warming (Baumgarten et al. 2026; Li et al. 2023). Species with indeterminate growth strategies may shift leaf senescence more flexibly under favourable conditions during late summer or the beginning of autumn. Thus, for these species, warming in late summer could sustain sink activity later into summer and autumn, thereby delaying carbon source–sink imbalance and senescence under warming.

By contrast, determinate growth species have a more fixed growth period and complete leaf production early (before the summer solstice for *A. platanoides* in our experiment). Once shoot, and radial growth cease, continued carbon assimilation under warming may accelerate source–sink imbalance and contribute to earlier senescence, potentially through sugar‐signalling pathways (Wingler et al. 2006). These species may follow a more fixed senescence programme, likely with a higher control of photoperiod. However, this hypothesis remains untested and deserves further investigations to improve our understanding of the effects of early‐ and late‐season warming on leaf senescence timing depending on species growth strategies.

### 4.4 Limitations of the Study

A first limitation is inherent to the experimental design, which imposes the need to conduct statistical inference of the treatment effect at the OTC level, with only two OTCs per treatment. This limited chamber‐level replication reduces the power to detect potential heterogeneity among chambers and therefore constrains the strength of treatment‐level inference. Nevertheless, pre‐solstice OTC effects on temperature, spring phenology, and growth were found to be small within the treatments (Figures S1 and S2, Tables S2 and S3), suggesting limited variation among the OTCs. While this does not fully resolve the lack of chamber‐level replication, it provides some support for the consistency of the observed patterns.

A second limitation lies in potential confounding factors between budburst timing and growth rate, as both can influence leaf senescence (Zohner et al. 2023; Wu et al. 2026). However, in our experiment *A. platanoides* exhibited a substantial advance in budburst under warming but no change in growth rate in terms of height or diameter increments, and we also observed no effect on twig NSC reserves and no shift in leaf senescence, in contrast to the other two species. This is consistent with our interpretation that senescence onset may be more closely linked to growth dynamics, sugar allocation, and developmental processes than budburst timing alone. The widespread carry‐over effect between spring and autumn phenology observed in extra‐tropical regions from both ground observations (Keenan and Richardson 2015) and remote sensing (Wu et al. 2026) may therefore primarily reflect the influence of earlier spring phenology on seasonal growth and tissue development (Zani et al. 2020). In addition, the stimulating effect of early‐season warming on growth was relatively modest in our study and limited to radial increment. Repeating the experiment over multiple years would be valuable, as early‐season warming can have carry‐over effects on subsequent‐year growth, as demonstrated experimentally in *Prunus spinosa* (Vander Mijnsbrugge et al. 2022).

Nutrient availability may also have constrained growth and developmental responses to warming in our experiment. Earlier budburst and warmer conditions in the warming treatment may have accelerated nutrient depletion and thereby limited even stronger growth responses. However, leaf nitrogen content tended to be higher in the warming treatment shortly after the summer solstice (Figure S5), suggesting that nitrogen did not limit the increased growth demand in the warming treatment. Water supply was not limiting, as irrigation was kept equal across treatments. Although reducing irrigation in the control could have accentuated treatment contrasts, we deliberately avoided any drought stress to exclude confounding effects on senescence, which may result from leaf scorching or desiccation rather than actual programmed senescence (Bergström et al. 2026).

NSCs were measured only once, around the summer solstice, and exclusively in apical twigs and leaves to avoid interfering with the leaf‐senescence process. This limited sampling restricts our ability to infer seasonal dynamics or whole‐plant carbon status. In particular, NSC concentrations in major storage pools such as coarse roots remain unknown. Assessing NSC allocation in these larger carbon reservoirs—ideally across the whole growing season—would provide valuable insights into how plants adjust carbon storage under stimulated growth conditions, which in turn could alter the onset of senescence. In addition, because twig NSC was measured 13 days after transferring saplings from the OTCs to a common greenhouse, the magnitude of treatment differences may have been underestimated. However, given the nearly 6‐month duration of the treatments, we expect relative differences in twig NSC concentration between treatments to have remained unaffected by this lag. Despite these limitations, our NSC measurements provide indicative patterns suggesting that early‐season warming altered source–sink carbon balance, most clearly in *T. cordata*. We encourage future experiments that manipulate growth rates to monitor NSC concentrations regularly in both source and sink organs from budburst to senescence and to test the relationship between growth, NSC dynamic and leaf senescence onset at both treatment and individual levels.

Finally, because seedlings or saplings are generally more opportunistic and phenologically more plastic than mature trees (Vitasse 2013), our results may underestimate the strength of the relationship between early‐summer growth and leaf senescence timing in adult trees. In mature trees, phenology and development typically rely more on internal controls and exhibit reduced flexibility to environmental variation (Körner 2012). At the same time, large storage pools in mature trees can buffer NSC fluctuations and modulate the source–sink balance. Therefore, monitoring their interannual growth dynamics (e.g., using dendrometers) together with NSC measurements and leaf senescence observations or leaf reflectance metrics represents a promising avenue to better understand these relationships.

## 5 Conclusion

Our study indicates that spring and early summer temperatures can have lasting effects on the onset of tree leaf senescence, likely linked to changes in growth dynamics and carbohydrate allocation. Warmer pre‐solstice conditions advanced the beginning of chlorophyll degradation and leaf discolouration in autumn in *C. betulus* and *T. cordata*, but not in *A. platanoides*. The fact that only species showing enhanced radial growth also exhibited earlier senescence suggests that growth rate, rather than budburst timing alone, may play an important role in shaping autumn phenology. Our results further indicate that the response of leaf senescence to warming may vary according to species' growth strategies—determinate versus indeterminate—although this pattern should be confirmed across a broader range of species. Finally, our findings are consistent with a potential role of growth‐mediated carbon dynamics in linking carry over effect of spring and early summer conditions to autumn phenology. Future studies integrating transcriptomics, seasonal changes in phytohormones (e.g. ABA and ethylene), and NSC dynamics under experimental warming will be needed to identify the molecular and physiological mechanisms linking early season growth and development to autumn leaf senescence.

## Supporting information


Supporting File 1

## Data Availability

Data supporting the findings of this study are available at: https://doi.org/10.16904/envidat.766.

## References

[pce70740-bib-0001] Asad, M. A. U. , Z. Yan , L. Zhou , X. Guan , and F. Cheng . 2024. “How Abiotic Stresses Trigger Sugar Signaling to Modulate Leaf Senescence?” Plant Physiology and Biochemistry 210: 108650. 10.1016/j.plaphy.2024.108650 38653095

[pce70740-bib-0002] Asim, M. , Q. Hussain , X. Wang , et al. 2022. “Mathematical Modeling Reveals That Sucrose Regulates Leaf Senescence via Dynamic Sugar Signaling Pathways.” International Journal of Molecular Sciences 23, no. 12: 6498. 10.3390/ijms23126498 PMC9223756 35742940

[pce70740-bib-0061] Baumgarten, F. , S. Aitken , Y. Vitasse , R. D. Guy , and E. Wolkovich . 2026. “(In)determinacy in Woody Plants: Limits and Opportunities for Timing Growth in a Changing Climate.” Ecology Letters 29, no. 6: e70435. 10.1111/ele.70435 PMC13291767 42338305

[pce70740-bib-0003] Bergström, M. , Z. Wu , C. Grossiord , and Y. Vitasse . 2026. “Distinguishing Leaf Scorching From Senescence Under Climate Extremes.” Nature Climate Change 16: 744–745. 10.1038/s41558-026-02682-1.

[pce70740-bib-0004] Buchanan‐Wollaston, V. 1997. “The Molecular Biology of Leaf Senescence.” Journal of Experimental Botany 48, no. 307: 181–199.

[pce70740-bib-0005] Büntgen, U. , A. Piermattei , P. J. Krusic , J. Esper , T. Sparks , and A. Crivellaro . 2022. “Plants in the UK Flower a Month Earlier Under Recent Warming.” Proceedings Biological Sciences 289, no. 1968: 20212456. 10.1098/rspb.2021.2456 PMC8808087 35105239

[pce70740-bib-0006] Dietze, M. C. , A. Sala , M. S. Carbone , et al. 2014. “Nonstructural Carbon in Woody Plants.” Annual Review of Plant Biology 65: 667–687. 10.1146/annurev-arplant-050213-040054 24274032

[pce70740-bib-0007] Ettinger, A. K. , S. Gee , and E. M. Wolkovich . 2018. “Phenological Sequences: How Early‐Season Events Define Those That Follow.” American Journal of Botany 105, no. 10: 1771–1780. 10.1002/ajb2.1174 30324664

[pce70740-bib-0008] Etzold, S. , F. Sterck , A. K. Bose , et al. 2022. “Number of Growth Days and Not Length of the Growth Period Determines Radial Stem Growth of Temperate Trees.” Ecology Letters 25, no. 2: 427–439. 10.1111/ele.13933 PMC9299935 34882952

[pce70740-bib-0009] Fischer, A. M. , K. M. Strassmann , M. Croci‐Maspoli , et al. 2022. “Climate Scenarios for Switzerland CH2018—Approach and Implications.” Climate Services 26: 100288.

[pce70740-bib-0010] Fu, Y. S. H. , M. Campioli , Y. Vitasse , et al. 2014. “Variation in Leaf Flushing Date Influences Autumnal Senescence and Next Year's Flushing Date in two Temperate Tree Species.” Proceedings of the National Academy of Sciences of the United States of America 111, no. 20: 7355–7360. 10.1073/pnas.1321727111 PMC4034254 24799708

[pce70740-bib-0011] Garnot, V. S. F. , L. Spafford , J. Lever , et al. 2025. “Deep Learning Meets Tree Phenology Modelling: Phenoformer Versus Process‐Based Models.” Methods in Ecology and Evolution 16, no. 7: 1489–1506.

[pce70740-bib-0012] Gill, A. L. , A. S. Gallinat , R. Sanders‐DeMott , et al. 2015. “Changes in Autumn Senescence in Northern Hemisphere Deciduous Trees: A Meta‐Analysis of Autumn Phenology Studies.” Annals of Botany 116, no. 6: 875–888. 10.1093/aob/mcv055 PMC4640124 25968905

[pce70740-bib-0013] Grossiord, C. , C. Bachofen , J. Gisler , E. Mas , Y. Vitasse , and M. Didion‐Gency . 2022. “Warming May Extend Tree Growing Seasons and Compensate for Reduced Carbon Uptake During Dry Periods.” Journal of Ecology 110, no. 7: 1575–1589.

[pce70740-bib-0014] Hallé, F. , R. A. Oldeman , and P. B. Tomlinson . 1978. Tropical Trees and Forests: An Architectural Analysis. Springer.

[pce70740-bib-0015] Hoch, G. , A. Richter , and C. Körner . 2003. “Non‐Structural Carbon Compounds in Temperate Forest Trees.” Plant, Cell & Environment 26, no. 7: 1067–1081.

[pce70740-bib-0016] Keenan, T. F. , and A. D. Richardson . 2015. “The Timing of Autumn Senescence is Affected by the Timing of Spring Phenology: Implications for Predictive Models.” Global Change Biology 21, no. 7: 2634–2641. 10.1111/gcb.12890 25662890

[pce70740-bib-0017] Kikuzawa, K. 1984. “Leaf Survival of Woody Plants in Deciduous Broad‐Leaved Forests. 2. Small Trees and Shrubs.” Canadian Journal of Botany 62, no. 12: 2551–2556.

[pce70740-bib-0018] Klein, T. , Y. Vitasse , and G. Hoch . 2016. “Coordination Between Growth, Phenology and Carbon Storage in Three Coexisting Deciduous Tree Species in a Temperate Forest.” Tree Physiology 36, no. 7: 847–855. 10.1093/treephys/tpw030 27126226

[pce70740-bib-0019] Körner, C. 2003. Alpine Plant Life. 2nd ed. Springer.

[pce70740-bib-0020] Körner, C. 2012. Alpine Treelines. Springer.

[pce70740-bib-0021] Kozlowski, T. T. 1964. “Shoot Growth in Woody Plants.” Botanical Review 30, no. 3: 335–392.

[pce70740-bib-0022] Kozlowski, T. T. , and S. G. Pallardy . 1997. Growth Control in Woody Plants. Elsevier.

[pce70740-bib-0023] Li, X. , X. Wang , Y. Fang , et al. 2023. “Phenology Advances Uniformly in Spring but Diverges in Autumn Among Three Temperate Tree Species in Response to Warming.” Agricultural and Forest Meteorology 336: 109475.

[pce70740-bib-0024] Lihavainen, J. , E. Edlund , L. Björkén , P. Bag , K. M. Robinson , and S. Jansson . 2021. “Stem Girdling Affects the Onset of Autumn Senescence in Aspen in Interaction With Metabolic Signals.” Physiologia Plantarum 172, no. 1: 201–217. 10.1111/ppl.13319 PMC8248097 33368469

[pce70740-bib-0025] Lim, P. O. , H. J. Kim , and H. Gil Nam . 2007. “Leaf Senescence.” Annual Review of Plant Biology 58: 115–136. 10.1146/annurev.arplant.57.032905.105316 17177638

[pce70740-bib-0026] Luo, T. , X. Liu , L. Zhang , X. Li , Y. Pan , and I. J. Wright . 2018. “Summer Solstice Marks a Seasonal Shift in Temperature Sensitivity of Stem Growth and Nitrogen‐Use Efficiency in Cold‐Limited Forests.” Agricultural and Forest Meteorology 248: 469–478.

[pce70740-bib-0027] Luo, Y. , C. Zohner , and T. W. Crowther , et al. 2026. “Internal Physiological Drivers of Leaf Development in Trees: Understanding the Relationship Between Non‐Structural Carbohydrates and Leaf Phenology.” Functional Ecology 40: 1151–1164.

[pce70740-bib-0028] Martínez‐Vilalta, J. , A. Sala , D. Asensio , et al. 2016. “Dynamics of Non‐Structural Carbohydrates in Terrestrial Plants: A Global Synthesis.” Ecological Monographs 86, no. 4: 495–516.

[pce70740-bib-0029] Maurya, J. P. , and R. P. Bhalerao . 2017. “Photoperiod‐ and Temperature‐Mediated Control of Growth Cessation and Dormancy in Trees: A Molecular Perspective.” Annals of Botany 120, no. 3: 351–360. 10.1093/aob/mcx061 PMC5591416 28605491

[pce70740-bib-0030] Meier, M. , C. Bigler , and I. Chuine . 2025. “Aging and Stress Explain the Earlier Start of Leaf Senescence in Trees in Warmer Years: Translating the Latest Findings on Senescence Regulation Into the DP3 Model (v1.1).” Geoscientific Model Development 18, no. 19: 6963–6985.

[pce70740-bib-0031] Meng, F. , A. J. Felton , J. Mao , et al. 2024. “Consistent Time Allocation Fraction to Vegetation Green‐Up Versus Senescence Across Northern Ecosystems Despite Recent Climate Change.” Science Advances 10, no. 23: eadn2487. 10.1126/sciadv.adn2487 PMC11160464 38848369

[pce70740-bib-0032] Menzel, A. , Y. Yuan , M. Matiu , et al. 2020. “Climate Change Fingerprints in Recent European Plant Phenology.” Global Change Biology 26, no. 4: 2599–2612. 10.1111/gcb.15000 31950538

[pce70740-bib-0033] Millard, P. , and G. Grelet . 2010. “Nitrogen Storage and Remobilization by Trees: Ecophysiological Relevance in a Changing World.” Tree Physiology 30, no. 9: 1083–1095. 10.1093/treephys/tpq042 20551251

[pce70740-bib-0034] Ono, K. , Y. Nishi , A. Watanabe , and I. Terashima . 2001. “Possible mechanisms of adaptive leaf senescence.” Plant Biology 3, no. 3: 234–243.

[pce70740-bib-0035] Panchen, Z. A. , R. B. Primack , A. S. Gallinat , et al. 2015. “Substantial Variation in Leaf Senescence Times Among 1360 Temperate Woody Plant Species: Implications for Phenology and Ecosystem Processes.” Annals of Botany 116, no. 6: 865–873. 10.1093/aob/mcv015 PMC4640117 25808654

[pce70740-bib-0036] Piao, S. , Q. Liu , A. Chen , et al. 2019. “Plant Phenology and Global Climate Change: Current Progresses and Challenges.” Global Change Biology 25, no. 6: 1922–1940. 10.1111/gcb.14619 30884039

[pce70740-bib-0037] Rolland, F. , E. Baena‐Gonzalez , and J. Sheen . 2006. “Sugar Sensing and Signaling in Plants: Conserved and Novel Mechanisms.” Annual Review of Plant Biology 57: 675–709. 10.1146/annurev.arplant.57.032905.105441 16669778

[pce70740-bib-0038] Silvestro, R. , A. Deslauriers , and P. Prislan , et al. 2025. “From Roots to Leaves: Tree Growth Phenology in Forest Ecosystems.” Current Forestry Reports 11, no. 1: 12.

[pce70740-bib-0039] Spafford, L. , J. J. Lever , A. Gessler , et al. 2025. “Stability and Transferability of Broadly Trained Phenology Models in a Changing Climate.” Agricultural and Forest Meteorology 372: 110685.

[pce70740-bib-0040] Thomas, H. 2013. “Senescence, Ageing and Death of the Whole Plant.” New Phytologist 197, no. 3: 696–711. 10.1111/nph.12047 23176101

[pce70740-bib-0041] Vander Mijnsbrugge, K. , J. M. Malanguis , S. Moreels , A. Turcsán , N. Van der Schueren , and E. Notivol Paino . 2022. “Direct Phenological Responses but Later Growth Stimulation Upon Spring and Summer/Autumn Warming of *Prunus spinosa* L. in a Common Garden Environment.” Forests 13, no. 1: 23.

[pce70740-bib-0042] Vitasse, Y. 2013. “Ontogenic Changes Rather Than Difference in Temperature Cause Understory Trees to Leaf Out Earlier.” New Phytologist 198, no. 1: 149–155. 10.1111/nph.12130 23347086

[pce70740-bib-0043] Vitasse, Y. , C. François , N. Delpierre , et al. 2011. “Assessing the Effects of Climate Change on the Phenology of European Temperate Trees.” Agricultural and Forest Meteorology 151, no. 7: 969–980.

[pce70740-bib-0044] Vitasse, Y. , G. Hoch , C. F. Randin , et al. 2013. “Elevational Adaptation and Plasticity in Seedling Phenology of Temperate Deciduous Tree Species.” Oecologia 171, no. 3: 663–678. 10.1007/s00442-012-2580-9 23306445

[pce70740-bib-0045] Vitasse, Y. , F. Baumgarten , C. M. Zohner , et al. 2021. “Impact of Microclimatic Conditions and Resource Availability on Spring and Autumn Phenology of Temperate Tree Seedlings.” New Phytologist 232, no. 2: 537–550. 10.1111/nph.17606 PMC8518844 34235742

[pce70740-bib-0046] Vitasse, Y. , F. Baumgarten , C. M. Zohner , et al. 2022. “The Great Acceleration of Plant Phenological Shifts.” Nature Climate Change 12, no. 4: 300–302.

[pce70740-bib-0047] Vitasse, Y. , A. Lenz , G. Hoch , and C. Körner . 2014. “Earlier Leaf‐Out Rather Than Difference in Freezing Resistance Puts Juvenile Trees at Greater Risk of Damage Than Adult Trees.” Journal of Ecology 102, no. 4: 981–988.

[pce70740-bib-0048] Wang, P. , C. Fu , L. Wang , and T. Yan . 2022. “Delayed Autumnal Leaf Senescence Following Nutrient Fertilization Results in Altered Nitrogen Resorption.” Tree Physiology 42, no. 8: 1549–1559. 10.1093/treephys/tpac028 35274706

[pce70740-bib-0049] Wingler, A. , and T. Roitsch . 2008. “Metabolic Regulation of Leaf Senescence: Interactions of Sugar Signalling With Biotic and Abiotic Stress Responses.” Plant Biology 10, no. s1: S50–S62. 10.1111/j.1438-8677.2008.00086.x 18721311

[pce70740-bib-0050] Wingler, A. , and R. Henriques . 2022. “Sugars and the Speed of Life—Metabolic Signals That Determine Plant Growth, Development and Death.” Physiologia Plantarum 174, no. 2: e13656. 10.1111/ppl.13656 PMC9314607 35243645

[pce70740-bib-0051] Wingler, A. , S. Purdy , J. A. MacLean , and N. Pourtau . 2006. “The Role of Sugars in Integrating Environmental Signals During the Regulation of Leaf Senescence.” Journal of Experimental Botany 57, no. 2: 391–399. 10.1093/jxb/eri279 16157653

[pce70740-bib-0052] Wu, Z. , Y. H. Fu , and T. W. Crowther , et al. 2026. “Carry‐Over Effects Between Spring and Autumn Phenology Differ Among the World's Biomes.” National Science Review 13: nwag082. 10.1093/nsr/nwag082 PMC12993445 41853091

[pce70740-bib-0053] Xie, Y. , X. Wang , A. M. Wilson , and J. A. Silander Jr. 2018. “Predicting Autumn Phenology: How Deciduous Tree Species Respond to Weather Stressors.” Agricultural and Forest Meteorology 250–251: 127–137.

[pce70740-bib-0054] Yoshida, S. 2003. “Molecular Regulation of Leaf Senescence.” Current Opinion in Plant Biology 6, no. 1: 79–84. 10.1016/s1369526602000092 12495755

[pce70740-bib-0055] Yu, H. , Q. Yao , L. Qiu , Z. Mo , and T. Tan . 2025. “Earlier End of Photosynthesis Driven by Advanced Photosynthetic Onset and Elevated Early‐Season Temperatures.” Environmental Research Letters 20: 124021.

[pce70740-bib-0056] Zahnd, C. , M. Zehnder , M. Arend , A. Kahmen , and G. Hoch . 2024. “Uniform Carbon Reserve Dynamics Along the Vertical Light Gradient in Mature Tree Crowns.” Tree Physiology 44, no. 13: 232–245. 10.1093/treephys/tpae005 PMC11898625 38198739

[pce70740-bib-0057] Zani, D. , T. W. Crowther , L. Mo , S. S. Renner , and C. M. Zohner . 2020. “Increased Growing‐Season Productivity Drives Earlier Autumn Leaf Senescence in Temperate Trees.” Science 370, no. 6520: 1066–1071. 10.1126/science.abd8911 33243884

[pce70740-bib-0058] Zhang, Y.‐L. , A. Gessler , M. M. Lehmann , et al. 2025. “Exogenous Sugar Addition Can Exacerbate Root Carbon Limitation in Trees.” New Phytologist 247, no. 2: 577–594. 10.1111/nph.70231 40400220

[pce70740-bib-0059] Zohner, C. M. , L. Mirzagholi , S. S. Renner , et al. 2023. “Effect of Climate Warming on the Timing of Autumn Leaf Senescence Reverses After the Summer Solstice.” Science 381, no. 6653: eadf5098. 10.1126/science.adf5098 37410847

[pce70740-bib-0060] Zuccarini, P. , N. Delpierre , B. Mariën , J. Peñuelas , T. Heinecke , and M. Campioli . 2023. “Drivers and Dynamics of Foliar Senescence in Temperate Deciduous Forest Trees at Their Southern Limit of Distribution in Europe.” Agricultural and Forest Meteorology 342: 109716.

