# Supplementary material for: Early‐Season Warming Advances Autumn Senescence in Temperate Tree Saplings in Association With Early Growth Dynamics
Source: Plant Cell Environ. 2026 Aug 5;49(11):8980–92. doi: 10.1111/pce.70740 (PMC13646692; doi:10.1111/pce.70740)
Supplement: Supplementary file 1 [file PCE-49-8980-s001.docx]

## *Plant, Cell & Environment* Supporting Information

Article title: Early-season warming advances autumn senescence in association with early-season growth dynamics

Yann Vitasse^1,2^, Shilong Ren^3^, Charlotte Grossiord^4,5^, Matthias Saurer^1^, Yunpeng Luo^1^, Yan-Li Zhang^1^, Zhaofei Wu^1^, Constantin Zohner^6^

The following Supporting Information is available for this article (short titles):

**Table S1.** Provenances and initial height and diameter at the beginning of the experiment.

**Table S2.** Model-estimated effect of pre-solstice warming on leaf-out timing for each species.

**Table S3.** Model-estimated effect of pre-solstice warming on pre-solstice and post-solstice diameter and height increments.

**Table S4.** Model-estimated effect of pre-solstice warming on NSC concentrations (free sugars and starch) in twigs and leaves around summer solstice for each species.

**Table S5.** Model-estimated effect of pre-solstice warming on maximum carbon assimilation (Amax) measured before or after the summer solstice for each species.

**Table S6.** Effects of pre-solstice warming on leaf senescence dynamics for each species.

**Fig. S1.** Leaf-out timing of the three species in the ambient and warming OTCs.

**Fig. S2.** Growth increment during pre-solstice conditions in the ambient and warming OTCs.

**Fig. S3.** Maximum carbon assimilation in saplings exposed to pre-solstice warming or control conditions, measured either before (one date) or after (three dates) the summer solstice.

**Fig. S4.** Progression of chlorophyll index values during senescence for each species as a function of the pre-solstice treatment (ambient and warming OTCs).

**Fig. S5.** Leaf carbon and nitrogen concentration shortly after the summer solstice.

**Fig. S6.** Correlations between early-season growth and autumn senescence

**Table S1.** Species provenances and initial height and diameter at the beginning of the experiment. N, numbers of plant used in the analyses.

| *Species* | *Provenance / altitude (m)* | *Initial height (mean ±SD, cm)* | *Initial stem collar diameter (mean±SD, mm)* | *n* |
| --- | --- | --- | --- | --- |
| *A. platanoides* | Zurich Hausen am Albis 650-690m | *64.7±19.1* | *6.22±1.26* | *39* |
| *C. betulus* | *Zurich Birmensdorf 550m* | *56.9±11.1* | *6.56±0.85* | *46* |
| *T. cordata* | *Saint-Gallen Quarten 460-640m* | *56.9±16* | *6.96±1.32* | *48* |

**Table S2.** Estimated effects of warming on leaf-out date for each species based on linear mixed-effects models including the pre-solstice OTC as a random intercept and species, treatment (warming vs. control) and their interactions as fixed effects. Values represent species-specific differences in the marginal means between warming and control treatments (Warming − Control), with associated standard errors (SE), t-values, degrees of freedom (df), and p-values. Degrees of freedom were calculated using the Kenward–Roger approximation. Variance attributed to pre-solstice OTC (random effect) and its proportion of total variance are shown for the full model.

| *Species* | *Warming-Control (days)* | *SE* | *t-value* | *df* | *p-value* | *Variance OTC* | *Variance OTC (%)* |
| --- | --- | --- | --- | --- | --- | --- | --- |
| *A. platanoides* | -20.2 | 2.1 | 9.5 | 24.9 | **<0.0001** | 0 | 0 |
| *C. betulus* | -14.8 | 1.8 | 8.2 | 14.5 | **<0.0001** |  |  |
| *T. cordata* | -28.1 | 1.8 | 15.5 | 14.5 | **<0.0001** |  |  |

**Table S3.** Estimated effects of warming on pre-solstice and post-solstice diameter and height increments for each species based on linear mixed-effects models including the pre-solstice OTC as a random intercept and species, treatment (warming vs. control) and their interactions as fixed effects. Values represent species-specific differences in the marginal means between warming and control treatments (Warming − Control), with associated standard errors (SE), t-values, degrees of freedom (df), and p-values. Degrees of freedom were calculated using the Kenward–Roger approximation. Variance attributed to pre-solstice OTC (random effect) and its proportion of total variance are shown for the full model.

|  | *Species* | *Warming-Control* | *SE* | *t-value* | *df* | *p-value* | *Variance OTC* | *Variance OTC (%)* |
| --- | --- | --- | --- | --- | --- | --- | --- | --- |
| *Height increment before summer solstice (cm)* | | | |  |  |  |  |  |
|  | *A. platanoides* | -1.6 | 3.6 | 0.44 | 19.9 | 0.664 | 0.0 | 0.0 |
|  | *C. betulus* | +2.7 | 3.3 | -0.82 | 15.8 | 0.423 |  |  |
|  | *T. cordata* | -1.4 | 3.3 | 0.41 | 15.8 | 0.689 |  |  |
| *Diameter increment before summer solstice (mm)* | | |  |  |  |  |  |  |
|  | *A. platanoides* | +0.0 | 0.2 | -0.15 | 7.9 | 0.887 | 0.01 | 3.3 |
|  | *C. betulus* | **+0.7** | **0.2** | **-3.29** | **7.1** | **0.013** |  |  |
|  | *T. cordata* | **+1.0** | **0.2** | **-4.64** | **7.1** | **0.002** |  |  |
| *Height increment after summer solstice (cm)* | | | |  |  |  |  |  |
|  | *A. platanoides* | +1.0 | 3.9 | -0.27 | 8.9 | 0.796 | 3.63 | 2.9 |
|  | *C. betulus* | -1.1 | 3.7 | 0.31 | 7.4 | 0.768 |  |  |
|  | *T. cordata* | -3.2 | 3.7 | 0.87 | 7.4 | 0.413 |  |  |
| *Diameter increment after summer solstice (mm)* | | |  |  |  |  |  |  |
|  | *A. platanoides* | -0.3 | 0.3 | 0.9 | 5.5 | 0.404 | 0.03 | 6.3 |
|  | *C. betulus* | +0.0 | 0.3 | -0.06 | 5.0 | 0.955 |  |  |
|  | *T. cordata* | +0.1 | 0.3 | -0.46 | 5.0 | 0.663 |  |  |

**Table S4.** Estimated effects of warming on NSC concentrations (free sugars and starch) in twigs and leaves around summer solstice for each species based on linear models including species treatment (warming vs. control) and their interactions as fixed effects. Values represent species-specific differences in the marginal means between warming and control treatments (Warming − Control), with associated standard errors (SE), t-values, degrees of freedom (df), and p-values. Degrees of freedom were calculated using the Kenward–Roger approximation.

|  | *Species* | *Warming-Control* | *SE* | *t-value* | *df* | *p-value* |
| --- | --- | --- | --- | --- | --- | --- |
| *Twig, free sugars (%)* | | | |  |  |  |
|  | *A. platanoides* | +0.1 | 0.2 | -0.74 | 27 | 0.468 |
|  | *C. betulus* | NA | NA | NA | NA | NA |
|  | *T. cordata* | **-0.6** | **0.2** | **2.98** | **27** | **0.006** |
| *Twig, starch (%)* | | |  |  |  |  |
|  | *A. platanoides* | -0.4 | 0.8 | 0.44 | 27 | 0.666 |
|  | *C. betulus* | NA | NA | NA | NA | NA |
|  | *T. cordata* | **+3.1** | **0.9** | **-3.58** | **27** | **<0.001** |
| *Leaves, free sugars (%)* | | | |  |  |  |
|  | *A. platanoides* | **-1.9** | **0.6** | **3.32** | **41** | **0.002** |
|  | *C. betulus* | +1.0 | 0.5 | -1.82 | 41 | 0.076 |
|  | *T. cordata* | **-1.5** | **0.5** | **2.73** | **41** | **0.009** |
| *Leaves, starch (%)* | | |  |  |  |  |
|  | *A. platanoides* | -0.6 | 0.6 | 1.02 | 41 | 0.313 |
|  | *C. betulus* | **-2.1** | **0.6** | **3.65** | **41** | **<0.001** |
|  | *T. cordata* | +0.3 | 0.6 | -0.58 | 41 | 0.568 |

**Table S5.** Estimated effects of warming on maximum carbon assimilation (Amax) measured before or after the summer solstice based on linear mixed-effects models including the pre-solstice OTC as random intercept and species, treatment (warming vs. control) and their interactions as fixed effects. Values represent species-specific differences in the marginal means between warming and control treatments (Warming − Control), with associated standard errors (SE), t-values, degrees of freedom (df), and p-values. Degrees of freedom were calculated using the Kenward–Roger approximation. Variance attributed to pre-solstice OTC (random effect) and its proportion of total variance are shown for the full model.

|  | *Species* | *Warming-Control* | *SE* | *t-value* | *df* | *p-value* | *Variance OTC* | *Variance OTC (%)* |
| --- | --- | --- | --- | --- | --- | --- | --- | --- |
| *Assimilation before summer solstice (μmol m^−2^ s^−1^)* | | | |  |  |  |  |  |
|  | *A. platanoides* | -0.03 | 1.03 | 0.03 | 59.4 | 0.98 | 0.086 | 2.9 |
|  | *C. betulus* | +0.40 | 1.03 | -0.39 | 59.4 | 0.69 |  |  |
|  | *T. cordata* | -0.19 | 1.03 | 0.19 | 59.4 | 0.85 |  |  |
| *Assimilation after summer solstice (μmol m^−2^ s^−1^)* | | |  |  |  |  |  |  |
|  | *A. platanoides* | -0.28 | 0.65 | 0.43 | 12.8 | 0.674 |  |  |
|  | *C. betulus* | **-2.04** | **0.65** | **3.15** | **12.8** | **0.008** |  |  |
|  | *T. cordata* | -0.47 | 0.64 | 0.73 | 15.0 | 0.477 |  |  |

**Table S6.** Effects of pre-solstice warming on leaf senescence dynamics for each species, estimated from species-specific GAMMs. Values represent the parametric effect of pre-solstice warming  (β ± SE) on leaf discoloration (%) or chlorophyll index averaged across the whole monitoring period. Negative values of leaf discoloration or positive values of chlorophyll index indicate **faster progression of senescence** under warming. Significance was assessed using the parametric treatment term in species-specific GAMMs. Standard errors (SE), t-values, p-values, and adjusted R² are shown.

|  | Species | β (%) | SE | t-value | p-value | Adj. R2 |
| --- | --- | --- | --- | --- | --- | --- |
| Leaf discoloration (%) | | | |  |  |  |
|  | *A. platanoides* | -2.2 | 2.5 | -0.87 | 0.38 | 0.89 |
|  | *C. betulus* | **-5.1** | **1.2** | **-4.36** | **<0.001** | **0.89** |
|  | *T. cordata* | **-4.1** | **0.9** | **-4.36** | **<0.001** | **0.91** |
| Chlorophyll index | | | |  |  |  |
|  | *A. platanoides* | 0.88 | 0.5 | 1.81 | 0.071 | 0.83 |
|  | *C. betulus* | 0.56 | 0.3 | 1.90 | 0.057 | 0.80 |
|  | *T. cordata* | **0.97** | **0.3** | **3.50** | **<0.001** | **0.84** |

**Fig. S1.**

**Fig. S1.** Leaf-out date (stage 3) of the three species in the two ambient and two warming OTCs. Boxplots show the median, first and third quartiles and whiskers extending to 1.5 times the interquartile range. C1, Control chamber 1 at ambient temperature; C2, Control chamber 2 at ambient temperature; W1, Warming chamber 1 at +5°C above the ambient; W2, Warming chamber 2 at +5°C above the ambient.

**Fig. S2.**

**Fig. S2.** Growth increment in height (A) and diameter (B) from budburst to the summer solstice in control and warming treatments for each of the four chambers used. Boxplots show the median, first and third quartiles and whiskers extending to 1.5 times the interquartile range.

**Fig. S3**

**Fig. S3.** Maximum assimilation measured in the Control and Warming treatments before the summer solstice and monthly thereafter for three months. After the first measurement (13 June 2023), all plants were moved into the same greenhouse on 22 June 2023; thus, any differences observed after June may reflect the effects of the pre-solstice treatment. Boxplots show the median, first and third quartiles and whiskers extending to 1.5 times the interquartile range. Statistical significance of treatment effects was assessed using linear mixed-effects models with pre-solstice OTCs as a random effect. *, *P* <0.05; **, *P* <0.01.

**Fig. S4**

**Fig. S4.** Progression of chlorophyll index values during late summer and autumn for each species as a function of the pre-solstice treatment (ambient and warming OTCs). Left panel: dots represent mean chlorophyll index values at each date, and lines represent the modelled progression using mixed-effect generalized additive models (GAMMs), with day of year as the smoothing term and climate chamber as a random effect. Shaded areas indicate 95% confidence intervals of the GAMMs fits. Vertical dashed lines in the left panel mark the day of year when 20%, 50%, and 80% of modeled discoloration occurred in the warming treatment (see Fig. 6). Right panel: chlorophyll index values corresponding to these three dates, with associated 95% confidence intervals derived from the GAMMs. n = 17–24 per species and treatment at each monitoring date.

**Fig. S5.**

**Fig. S5.** Leaf carbon and nitrogen concentration shortly after the summer solstice (28 June 2023). (A) Carbon-to-nitrogen (C:N) ratio, (B) Carbon concentration (% of dry mass), and (C) Nitrogen concentration (% of dry mass). Boxplots show the median, first and third quartiles and whiskers extending to 1.5 times the interquartile range. Statistical significance of treatment effects was assessed using linear models. *, *P* <0.05; **, *P* <0.01.

**Fig. S6.** Relationship between early-season diameter increment and leaf chlorophyll index and coloration at the beginning of senescence (DOY 290). Points represent individual trees from the presolstice warming (red) or control (blue). Black lines show linear regressions (solid: p < 0.05; dashed: p ≥ 0.05). Shaded areas indicate 95% confidence intervals. Reported are Pearson’s r, p-value, and sample size (n).
